# Case Report: Early Treatment With Chenodeoxycholic Acid in Cerebrotendinous Xanthomatosis Presenting as Neonatal Cholestasis

**DOI:** 10.3389/fped.2020.00382

**Published:** 2020-07-16

**Authors:** Irene Degrassi, Chiara Amoruso, Giuseppe Giordano, Marina Del Puppo, Andrea Mignarri, Maria Teresa Dotti, Mauro Naturale, Gabriella Nebbia

**Affiliations:** ^1^Pediatric Intermediate Care Unit, Fondazione IRCCS Ca' Granda Ospedale Maggiore Policlinico, Milan, Italy; ^2^Mass Spectrometry Laboratory, Women's and Children' Health Department, University of Padua, Fondazione Istituto di Ricerca Pediatrica Città della Speranza, Padova, Italy; ^3^School of Medicine and Surgery, University of Milano-Bicocca, Milan, Italy; ^4^Unit of Neurology and Neurometabolic Diseases, Departement of Neurological and Motor Sciences, University of Siena, Siena, Italy

**Keywords:** inborn errors of bile acid metabolism, cerebrotendinous xanthomatosis, neonatal cholestasis, chenodeoxycholic acid, CYP27A1 gene, cholestanol

## Abstract

**Background:** Cerebrotendinous xanthomatosis (CTX) is an inborn disorder of bile acid synthesis which causes progressive accumulation of toxic metabolites in various organs, particularly in brain and tendons. Most cases are diagnosed and treated in the second or third decade of life, when neurological involvement appears. We describe a case of CTX presenting as neonatal cholestasis.

**Results:** The child presented cholestasis at 2 months of life. In the following months jaundice slowly disappeared, with a normalization of bilirubin and aminotransferases, respectively, at 6 and 8 months. A LC-Mass Spectrometry of the urines showed the presence of cholestanepentols glucuronide, which led to the suspicion of cerebrotendinous xanthomatosis. The diagnosis was confirmed by the dosage of cholestanol in serum and the molecular genetic analysis of the *CYP27A1* gene. Therapy with chenodeoxycholic acid (CDCA) was started at 8 months and is still ongoing. The child was monitored for 13 years by dosage of serum cholestanol and urinary cholestanepentols. A strictly biochemical and neurological follow up was performed and no sign of neurological impairment was observed.

**Conclusions:** Prompt diagnosis and treatment of CTX presenting as neonatal cholestasis may prevent further neurological impairment.

## Introduction

**Figure 1:**
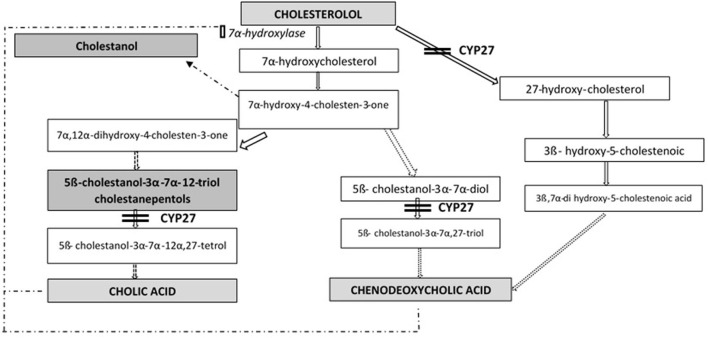
Bile acid synthesis in cerebrotendinous xanthomatosis.

Cerebrotendinous xanthomatosis (CTX) is an autosomal recessive lipid storage disorder with a defect in bile acid synthesis (1, 2). It is caused by mutations in gene *CYP27A1* (OMIM^*^606530), that encodes the mitochondrial sterol 27-hydroxylase enzyme (EC 1.14.13.15), catalyzing the first step in the process of cholesterol side chain oxidation. The synthesis of primary bile acids cholic acid (CA) and chenodeoxycholic acid (CDCA) is impaired: as a result, the normal feedback inhibition of 7α-hydroxylase is disrupted with consequent production of toxic metabolites (mainly cholestanol) and bile alcohol glucuronides, detectable, respectively, in plasma and urines (Figure 1). Cholestanol and cholesterol accumulate in particular in nervous system, eyes and tendons (3).

The estimated prevalence of CTX is 1:50.000 (4), with high variability among patients regarding age of onset and type of symptoms, since clinical manifestations appear along with the progressive accumulation of toxic metabolites (5). In infancy CTX may cause cholestatic jaundice, that usually spontaneously resolves (6, 7), so that the disease may be overlooked. During childhood cataract, chronic diarrhea and psychomotor retardation can be the first manifestations (8, 9); however, as the previous symptoms are aspecific, the diagnosis is often delayed until the appearance in adulthood of tendon xanthomas and the onset of neurological symptoms (spastic paresis, cerebellar ataxia, polyneuropathy, cognitive difficulties, dementia) (10).

Therapy with primary bile acids (chenodeoxycholic acid and cholic acid) is the standard treatment: it replaces the deficiency of primary bile acids and normalizes the levels of cholestanol, bile alcohols and cholesterol, with improvement in clinical, and biochemical manifestations (11, 12).

Here, we describe a long term follow-up of a girl with CTX, presenting as neonatal cholestasis at 2 months of life, promptly treated with CDCA since the age of 8 months.

## Case Description

The patient was a female baby of caucasian origin, born after an uncomplicated pregnancy and delivery. Neonatal weight was 3.100 Kg. The parents were not consanguineous and did not present any ophthalmological, neurological, cardiac or hepatic disease. She was breast-fed with normal growth and development until the second month of life, when jaundice was noticed, so that the patient was referred to the Pediatric Department of our hospital. Physical examination showed no dysmorphic features, severe jaundice and a slightly firm liver, palpable at 2 cm from the costal edge. No history of steathorrea, dark urine or acholic stools was reported. Blood tests showed high levels of total (13.56 mg/dl) and conjungated bilirubin (8.79 mg/dl), elevation of transaminases (AST 473 UI/l; ALT 530 UI/l), with normal gamma-GT, alkaline phosphatase, serum bile acids and INR. Ophtalmoscopic examination and abdominal ultrasound scan were normal. According to current protocols in cholestatic patients (13), we have started early supplementation with vitamin A, D, E and K since the age of 2 months to avoid complications related to fat soluble vitamins deficiency. Taking into account clinical and biochemical findings, main causes of neonatal cholestasis were excluded.

Considering the clinical picture of unexplained prolonged cholestasis, a percutaneous liver biopsy was performed, showing aspecific features of idiopathic hepatocellular cholestasis such as pronounced lobular disarray, giant-cells, bile pigment within hepatocytes, with slightly enlarged portal tracts in the absence of bile duct proliferation.

Therapy with ursodeoxycholic acid (UDCA) was started at 3 months of age at the dosage of 15 mg/Kg. In the following months jaundice slowly disappeared, with a normalization of bilirubin and transaminases, respectively, at 6 and 8 months. At the age of 7 months in view of the presence of intrahepatic cholestasis with normal colored stools, absence of pruritus, normal serum bile acids and gGT, urinary bile acids profile was performed in the suspicion of bile acid synthesis defects. The analysis showed the presence of 5ß-cholestanol-3α-7α-12-triol (cholestanepentols glucuronide) at the value of 38,4 ratio/mM creat (normal value: 0,86 ratio/mM creat), while serum cholestanol had a value of 3,140 ± 55 μg/dl (normal control value 470 μg/dl); these findings allowed a suspicion of CTX. Mutation analysis of CYP27A1 showed homozygosity for a deletion of 1,9 Kb with loss of exon 7,8,9 on chromosome 2q33-qter, leading to a definite diagnosis.

**Figure 2:**
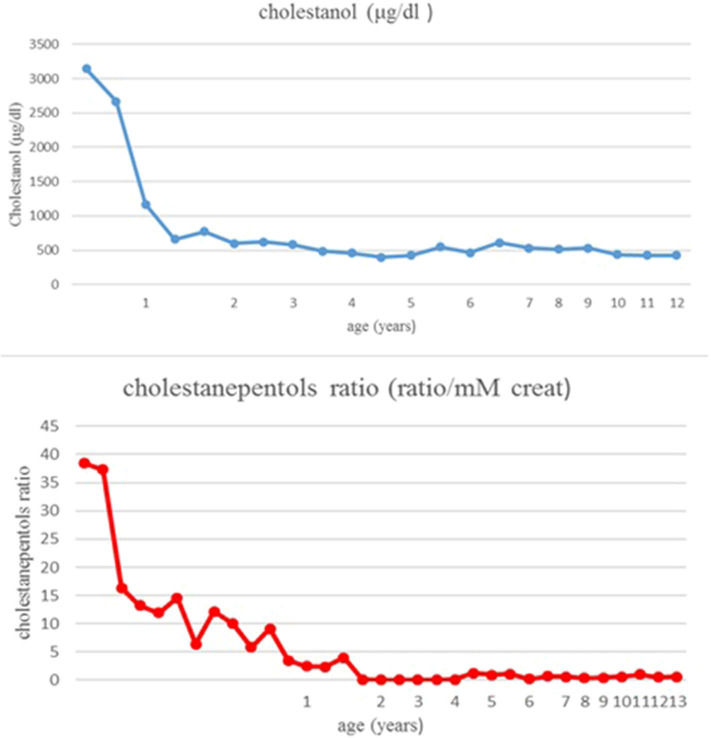
Cholestanol and cholestanepentols values during follow-up.

Therapy with UDCA was stopped and CDCA was started at 8 months of age. The initial dose was 10 mg/Kg/day, increased until 15 mg/Kg/day. A parallel, constant decrease of cholestanol in plasma was noticed: from 3,140 μg/dl to 600 μg/dl after 14 months of treatment (Figure 2). The patient has been followed for 13 years: in the absence of clinical manifestations and in the presence of normal laboratory values, the patient was strictly monitored with periodic assessment of plasma cholestanol and urinary cholestanepentols glucuronide to monitor silent accumulation of abnormal metabolites with consequent therapy adjustments (Figure 2). The values remained in the normal limits throughout the years and the therapy was well-tolerated, with no evidence of clinical or biochemical side effects. As regards hepatic involvement, serum liver function tests remained always in the normal limits. Periodic abdominal ultrasound scans were normal and transient elastography, performed at the age of eleven, showed normal liver stiffness (4.1 Kpa). Concerning possible neurologic involvement, annual electroencephalograms (EEG), neurological examinations and ophthalmoscopy controls were performed without detection of complications, as well as a brain magnetic resonance (MRI) performed at the age of 8 years. No xanthomas were detected in the Achilles tendons or other regions. Finally, the child showed a normal growth and cognitive development, with excellent school results and physical performance.

## Materials and Methods

### Urine Bile Acids

The qualitative and quantitative analysis was made by liquid chromatography-tandem mass spectrometry (LC-MS/MS). The urine sample used was calculated in base at 30 mg of creatinine (Cr) was considered consistent with effective suppression of the abnormal pathway. Urine samples were collected from the patients and stored at −20°C until analysis was carried out. The concentration of bile acids in the urine was corrected according to the Cr concentration and expressed as micromoles per millimole of Cr. For extraction of bile acids from urine we used “OASIS HLB” Waters solid-phase extraction cartridges (1 mL). The cartridges were prewashed with methanol and distilled water. The urine sample, previously normalized for the Cr concentration, was passed through a cartridge with 50 μL of internal standard (100 mM of CA and CDCA conjugated or not with glycine and taurine, labeled with four atoms of deuterium). The bile acids and alcohols were eluted with 400 μL of methanol, taken to dryness by a stream of N2, and then diluted with 100 μL of a solution of 50% acetonitrile. The sample was analyzed by the mass spectrometer micromass QUATTRO Ultima interfaced with HPLC HT Waters 2790 by electrospray ionization. The analysis was performed by direct injection (without a column). The negative ion mass spectra of the elutes were recorded in the mass range of 300 to 700 Da, which represents the mass ranges of the different urinary bile acids. Data were elaborated by neolynx software (MassLynx, Waters Corp, Milford, MA).

### Plasma Cholestanol

Serum levels of cholestanol were assayed as previously described (14). Briefly, deuterated cholestanol (1 μg) as internal standard was added to 0.2-mL serum samples and alkaline hydrolysis was carried out with 1 mL 1N NaOH in 90% ethanol at 60°C for 90 min under nitrogen. After addition of saline (1 mL), sterols were extracted with 2 mL petroleum ether and taken to dryness under a stream of nitrogen. The extracted sterols were then converted into their pentafluorobenzoyl derivatives (PFB), as described by (15), and purified by HPLC separation in order to remove excess cholesterol from lipid extract. The PFB-cholestanol fraction of each sample was collected and, after solvent evaporation, it was dissolved in 25 μl of toluene for GC-MS analysis. Analysis of sterols was carried out using a Thermo Finnigan GC-Q instrument (Waltham, MA, USA). For sterols quantification we focused specific ions: m/z 215 and 427 for PFB-cholestanol, m/z 219 and 431 for deuterated PFB-cholestanol. Calibration curves were prepared by spiking plasma with a fixed amount of internal standard and increasing amounts of cholestanol. These samples were treated and analyzed as the experimental samples. Concentrations were calculated on the basis of the slope of the standard curve and on the peak area ratio (sterol/IS) found in the sample. The assay results were linear (*r* > 0.98) in the tested ranges.

## Discussion

We describe a case of cerebrotendinous xanthomatosis presenting as neonatal cholestasis with normal levels of gammaGT in the first year of life. Other cholestatic infants with spontaneous recovery have been described (6, 16–18) and this benign clinical picture could explain why specific investigations for CTX are seldom performed, with an underdiagnosis in pediatric age. During the first months of life the alternative pathway of bile acid synthesis is important while the major pathway is immature. Since CYP27A1 is intensively involved in the alternative pathway, this may explain in part neonatal cholestasis (19). When the major pathway becomes mature, then cholestasis may resolve, as in our patient. Fatal neonatal cholestasis have been exceptionally reported (7, 20), but the presence of co-infection with CMV might have played an important role in this negative evolution.

The two primary bile acids (CDCA or CA) have been used as a treatment of this condition (5, 21), but most authors consider CDCA the treatment of choice, both in adults (4, 5, 22) and in children (8, 23). CA has been successfully used in some patients with CTX and in large cohorts of patients with other bile acid synthesis and zellweger spectrum disorders (12, 16, 24). Chenodeoxycholic acid seems to have a better biochemical profile as cholic acid levels are not reduced in CTX, while CDCA levels are deficient (25). Besides chenodeoxycholic acid has an effect on feedback inhibition of HMG CoA reductase, thereby suppressing cholesterol production and xanthomas' formation (26).

In an infant treated before the onset of cholestasis, CDCA has been reported as hepatotoxic (18). In this patient cholestasis developed after 6 weeks of treatment and liver enzymes returned to normal values within 3 months after cessation of CDCA. It must be noticed that jaundice with spontaneous resolution can be a manifestation of the disease, as in our patient, so that the relationship with the treatment is uncertain, in this patient hepatitis did not recur on re-challenge with CDCA (4, 18). Both CDCA and CA have been shown safe during pregnancy (5, 11, 27, 28).

In our patient CDCA was started at the age of 8 months; at this time her cholestanol level was already very high and dropped down under therapy. During the whole period of follow-up no further detection of abnormal urinary and plasma metabolites was noticed. In 13 years of follow-up the patient did not show any symptoms, with a completely normal neurological development, as well as persistently normal laboratory values; moreover, no collateral effect was noticed. We can hypothesize that the early beginning of the therapy, with rapid, persistent decrease of the toxic molecules, has prevented clinical complications. It has been previously demonstrated that starting treatment as early as possible can improve and even avoid the onset of neurological involvement (10, 29). Taking into account this observation, some authors suggest to include CTX in newborn screening (30, 31).

We suggest to look for the presence of cerebrotendinous xanthomatosis in patients with unexplained prolonged neonatal cholestasis with normal levels of gamma-GT and serum bile acids, in order to increase the rate and timing of new diagnoses and to start a prompt treatment. Since accumulation of toxic metabolites is progressive but may be clinically silent, we recommend a strict metabolic monitoring during the therapy with consequent dose adjustments. In conclusion, considering on one side the high frequency of severe clinical manifestations in CTX, and on the other side the availability of an effective and safe therapy, our experience stresses the importance of an early diagnosis and treatment, to prevent irreversible systemic and neurological damage.

## Data Availability Statement

Publicly available datasets were analyzed in this study. The raw data supporting the conclusions of this article will be made available by the authors, without undue reservation, to any qualified researcher.

## Ethics Statement

Ethical review and approval was not required for the study on human participants in accordance with the local legislation and institutional requirements. Written informed consent to participate in this study was provided by the participants' legal guardian/next of kin.

## Take Home Message

Cerebrotendinous xanthomatosis should be suspected in patients with unexplained neonatal cholestasis, even with spontaneous recovery. Considering on one side the high frequency of severe clinical manifestations in CTX, and on the other side the availability of an effective and safe therapy with CDCA, an early diagnosis and treatment is important to prevent irreversible systemic and neurological damage.

## Informed Consent

The patient's parents have provided written informed consent to genetic testing and publication of the case report.

## Author Contributions

ID and CA participated in the design of the work and wrote the draft of the article. AM and MTD contributed to the design of the work. GN designed the work and critically revised it. MDP performed plasma cholestarol analysis. GG and MN performed urinary bile acids profile. All authors have approved the final version of the article.

## Conflict of Interest

The authors declare that the research was conducted in the absence of any commercial or financial relationships that could be construed as a potential conflict of interest.

## Abbreviations

CA: cholic acid
CTX: cerebrotendinous xanthomatosis
CDCA: chenodeoxycholic acid
CYP27A1: sterol 27-hydroxylase
MS: Mass Spectrometry
SPE: solid phase extraction
FIA: flow injection analysis
UDCA: ursodeoxycholic acid
EEG: Electroencephalogram
MRI: magnetic resonance imaging.

## References

[1] Van Bogaert L Scherer HJ Froehlich A Epstein E Une deuxieme observation de cholesterinose tendineuse symetrique avec symptomes cerebraux. Ann Med. (1937) 42:69–101.

[2] Clayton PT. Disorders of bile acid synthesis. J Inherit Metab Dis. (2011) 34:593–604. 10.1007/s10545-010-9259-3 21229319

[3] Heubi JE Setchell KDR Bove KE. Inborn errors of bile acid metabolism. Clin Liver Dis. (2018) 22:671–87. 10.1016/j.cld.2018.06.006 30266156

[4] Verrips A Dotti MT Mignarri A Stelten BML Verma S Federico A. The safety and effectiveness of chenodeoxycholic acid treatment in patients with cerebrotendinous xanthomatosis: two retrospective cohort studies. Neurol Sci. (2019). 10.1007/s10072-019-04169-8 31863326 PMC7160076

[5] Salen G Steiner RED. Pidemiology diagnosis, and treatment of cerebrotendinous xanthomatosis (CTX). J Inherit Metab Dis. (2017) 40:771–81. 10.1007/s10545-017-0093-8 28980151

[6] Clayton PT Verrips A Sistermans E Mann A Mieli-Vergani G Wevers R. Mutations in the sterol 27-hydroxylase gene (CYP27A) cause hepatitis of infancy as well as cerebrotendinous xanthomatosis. J Inherit Metab Dis. (2002) 25:501–13. 10.1023/A:1021211520034 12555943

[7] Gong JY Setchell KDR Zhao J Zhang W Wolfe B Lu Y. Severe neonatal cholestasis in cerebrotendinous xanthomatosis: genetics, immunostaining, mass spectrometry. J Pediatr Gastroenterol Nutr. (2017) 65:561–8. 10.1097/MPG.0000000000001730 28937538

[8] Berginer VM Gross B Morad K Kfir N Morkos S Aaref S. Chronic diarrhea and juvenile cataracts: think cerebrotendinous xanthomatosis and treat. Pediatrics. (2009) 123:143–7. 10.1542/peds.2008-0192 19117873

[9] Degos B Nadjar Y Amador Mdel M Lamari F Sedel F Roze E. Natural history of cerebrotendinous xanthomatosis: a paediatric disease diagnosed in adulthood. Orphanet J Rare Dis. (2016) 16:41. 10.1186/s13023-016-0419-x 27084087 PMC4833925

[10] Mignarri A Gallus GN Dotti MT Federico A. A suspicion index for early diagnosis and treatment of cerebrotendinous xanthomatosis. J Inherit Metab Dis. (2014) 37:421–9. 10.1007/s10545-013-9674-3 24442603

[11] Yahalom G Tsabari R Molshatzki N Ephraty L Cohen H Hassin-Baer S. Neurological outcome in cerebrotendinous xanthomatosis treated with chenodeoxycholic acid: early versus late diagnosis. Clin Neuropharmacol. (2013) 36:78–83. 10.1097/WNF.0b013e318288076a 23673909

[12] Mandia D Chaussenot A Besson G Lamari F Castelnovo G Curot J. Cholic acid as a treatment for cerebrotendinous xanthomatosis in adults. J Neurol. (2019) 266:2043–50. 10.1007/s00415-019-09377-y 31115677

[13] Yang CH Perumpail BJ Yoo ER Ahmed A Kerner Jr J. Nutritional needs, and support for children with chronic liver disease. Nutrients. (2017) 9:1127 10.3390/nu9101127 29035331 PMC5691743

[14] Mignarri A Magni A Del Puppo Gallus GN Bjorkhem I Federico A. Evaluation of cholesterol metabolism in cerebrotendinous xanthomatosis. J Inherit Metab Dis. (2016) 39:75–83. 10.1007/s10545-015-9873-1 26153518

[15] Kuriyama M Fujiyama J Kasama T Osame M. High levels of plant sterols and cholesterol precursors in cerebrotendinous xanthomatosis. J Lipid Res. (1991) 32:223–9. 2066659

[16] Pierre G Setchell KDR Blyth J Preece MA Chakrapani A McKiernan P. Prospective treatment of cerebrotendinous xanthomatosis with cholic acid therapy. J Inherit metab Dis. (2008) 31 (Suppl. 2) (S241–5). 10.1007/s10545-008-0815-z 19125350

[17] Setchell KDR O' Connell N Russell DW A unique case of cerebrotendinous xanthomatosis presenting in infancy with cholestatic liver disease further highlights bile acid synthetic defects as an important category of metabolic liver disease (abstract). In: Falk Symposium 120. XVI International Bile Acid Meeting (2000).

[18] Huidekoper HH Vaz FM Verrips A Bosch AM. Hepatotoxicity due to chenodeoxycholic acid supplementation in an infant with cerebrotendinous xanthomatosis: implications for treatment. Eur J Pediatr. (2016) 175:143–6. 10.1007/s00431-015-2584-7 26156051 PMC4709371

[19] Setchell KD Dumaswala R Colombo C Ronchi M. Hepatic bile acid metabolism during early development revealed from the analysis of human fetal gallbladder bile. J Biol Chem. (1988) 263:16637–44. 3182806

[20] Von Bahr S Björkhem I Van't Hooft F Alvelius G Nemeth A Sjövall J. Mutation in the sterol 27-hydroxylase gene associated with fatal cholestasis in infancy. J Pediatr Gastroenterol Nutr. (2005) 40:481–6. 10.1097/01.MPG.0000150419.23031.2A 15795599

[21] Heubi JE Bove KE Setchell KDR. Oral cholic acid is efficacious and well tolerated in patients with bile acid synthesis and zellweger spectrum disorders. J Pediatr Gastroenterol Nutr. (2017) 65:321–26. 10.1097/MPG.0000000000001657 28644367 PMC5559188

[22] Duell PB Salen G Eichler FS DeBarber AE Connor SL Casaday L. Diagnosis, treatment, and clinical outcomes in 43 cases with cerebrotendinous xanthomatosis. J Clin Lipidol. (2018) 12:1169–78. 10.1016/j.jacl.2018.06.008 30017468

[23] Van Heijst AFJ Verrips A Wevers RA Cruysberg JR Renier WO Tolboom J. Treatment and follow-up of children with cerebrotendinous xanthomatosis. Eur J Pediatr. (1998) 157:313–6. 10.1007/s004310050818 9578968

[24] James E Heubi JE Setchell KDR. Open-label phase 3 continuation study of cholic acid in patients with inborn errors of bile acid synthesis. J Pediatr Gastroenterol Nutr. (2020) 70:423–9. 10.1097/MPG.0000000000002618 31899729

[25] Beppu T Seyama Y Kasama T Serizawa S Yamakawa T. Serum bile acid profiles in cerebrotendinous xanthomatosis. Clin Chim Acta. (1982) 118:167–75. 10.1016/0009-8981(82)90004-3 7055978

[26] Salen G Meriwether TW Nicolau G. Chenodeoxycholic acid inhibits increased cholesterol and cholestanol synthesis in patients with cerebrotendinous xanthomatosis. Biochem Med. (1975) 14:57–74. 10.1016/0006-2944(75)90020-4 1212241

[27] Gonzales E Matarazzo L Franchi-Abella S Dabadie A Cohen J Habes D. Cholic acid for primary bile acid synthesis defects: a life-saving therapy allowing a favorable outcome in adulthood. Orphanet J Rare Dis. (2018) 13:190. 10.1186/s13023-018-0920-5 30373615 PMC6206929

[28] Moghadasian MH Salen G Frohlich JJ Scudamore CH. Cerebrotendinous xanthomatosis: a rare disease with diverse manifestations. Arch Neurol. (2002) 59:527–9. 10.1001/archneur.59.4.527 11939886

[29] Stelten BML Huidekoper HH van de Warrenburg BPC Brilstra EH Hollak CEM Haak HR. Long-term treatment effect in cerebrotendinous xanthomatosis depends on age at treatment start. Neurology. (2019) 92:e83–e95. 10.1212/WNL.0000000000006731 30530799

[30] DeBarber AE Kalfon L Fedida A Fleisher Sheffer V Ben Haroush S Chasnyk N. Newborn screening for cerebrotendinous xanthomatosis is the solution for early identification and treatment. J Lipid Res. (2018) 59:2214–22. 10.1194/jlr.M087999 30135217 PMC6210902

[31] Vaz FM Bootsma AH Kulik W Verrips A Wevers RA Schielen PC. A newborn screening method for cerebrotendinous xanthomatosis using bile alcohol glucuronides and metabolite ratios. J Lipid Res. (2017) 58:1002–7. 10.1194/jlr.P075051 28314860 PMC5408618

